# Diverticular Pylephlebitis and Polymicrobial Septicemia

**DOI:** 10.1155/2017/6819474

**Published:** 2017-01-09

**Authors:** Pradhum Ram, Kamolyut Lapumnuaypol, Chitra Punjabi

**Affiliations:** Department of Internal Medicine, Albert Einstein Medical Center, Philadelphia, PA, USA

## Abstract

Diverticulitis primarily affects the sigmoid colon and is often complicated by intra-abdominal abscesses and fistulas. Rarely, however, mesenteric venous thrombosis has been known to occur. Optimal management is still unclear. We report the first case of polymicrobial sepsis resulting from diverticular pylephlebitis, managed successfully with bowel rest, antibiotics, and anticoagulation.

## 1. Introduction

Diverticulitis is caused by inflammation of out-pouches within the colonic wall, most commonly the sigmoid colon. Intra-abdominal abscesses, fistulas, and even diverticular perforation are not uncommon complications. Rarely, however, given severe inflammation, mesenteric venous thrombosis can occur, where a delay in diagnosis can result in significant morbidity [1–4].

## 2. Case

A 63-year-old male presented to the emergency department with fever and chills for four days. The patient's ability to provide a history was limited secondary to dementia and thus majority of the history was obtained from his wife. His past medical history was remarkable for hypertension, chronic obstructive pulmonary disease, and a remote history of a cerebrovascular accident with no residual motor weakness. His wife had noticed that he had been feeling increasingly weak over the past week prior to admission; however it was not until 3 days prior to admission when subjective fevers and chills were first noted. His review of systems was otherwise unremarkable and he denied any sick contacts. There was no reported history of weight loss or blood in the stool. In the ER, he was febrile to 39.2°C and tachycardic to 126. His physical exam was otherwise unremarkable.

Routine chemistry and hematology revealed white blood cell count of 26,500/*μ*L (polymorphonuclear cells 88% with bands 2%), platelet count of 69 × 10^3^/*μ*L, and lactate of 43 mg/dL (normal < 19 mg/dL). A chest X-ray and urine analysis were within normal limits. He was started on vancomycin, cefepime, and metronidazole. He was also fluid resuscitated. Blood cultures drawn were positive for gram positive cocci and gram negative rods within 18 hours of collection, which eventually speciated into* viridans streptococci* and* Prevotella oralis*. Following initial identification, a CT scan of the abdomen obtained showed pan colonic diverticulosis with a terminal ileal collection concerning diverticular phlegmon associated with extensive superior mesenteric vein thrombosis concerning an infected thrombophlebitis. The portal vein remained patent and a 2D ECHO was negative for endocarditis. He was initially started on continuous infusion of heparin and closely monitored for need for surgery. Clinical and laboratory parameters improved after 5 days of conservative management with a repeat CT abdomen showing improvement in the previously noted thrombophlebitis. He was then switched to therapeutic doses of enoxaparin, and given his clinical and radiological improvement he was discharged home to complete a 3-week course of amoxicillin-clavulanate. His white blood cell count on discharge was 14,900/*μ*L. The patient and his wife were given clear instructions to follow up with his primary care physician, as he would require a screening colonoscopy following resolution of his diverticular flare.

## 3. Discussion

Diverticulitis is defined as an inflammation of a diverticulum, which is not uncommonly accompanied by gross or microscopic perforation [1]. It is commonly a disease of the elderly and over 40% of patients in their 70s are thought to have diverticuli; although its exact pathogenesis remains unclear, complex interactions between colonic microbiota, colonic motility, inflammation on a microscopic level, and genetic factors are believed to play a part [2, 3].

Common complications of diverticulitis include perforation, abscess and phlegmon formation with perforation resulting in significant morbidity and mortality to patients. Mesenteric venous thrombosis (MVT) in our patient is a rarely reported complication of diverticulitis, with one epidemiologic study from Sweden reporting an incidence of MVT at less than 3 per 100,000 patients [4]. Conditions thought to be associated with MVT include trauma, pancreatitis, carcinoma, inflammatory bowel disease, portal hypertension, and congestive heart failure to name a few, with the underlying mechanism largely attributed to the prothrombotic state [5, 6].

Mesenteric venous thrombosis in the setting of intra-abdominal infection can be difficult to diagnose because of nonspecific nature of signs and symptoms as elucidated in our patient. Thus a high degree of suspicion is required in making the diagnosis, and contrast-enhanced CT plays a critical role in diagnosis with an accuracy of 90% [7]. Moreover, elderly patients with intra-abdominal sepsis can present with less symptoms and the mortality rate is also higher compared to younger patients [3]. Combining MVT in the setting of bacteremia, our patient also had pylephlebitis. There are only a few case reports where inferior mesenteric vein pylephlebitis has been reported in the setting of sigmoid diverticulitis; to our knowledge this would be the first reported case of polymicrobial sepsis associated with superior mesenteric vein thrombosis related to diverticulitis [8–11].

This condition can be safely managed medically if there is no evidence of peritonitis, bowel infarction, or perforation. The goal of treatment is to prevent intestinal infarction by maintaining adequate bowel perfusion. Some degree of controversy exists with regard to the need for anticoagulation in the absence of portal vein thrombus. In our patient intravenous unfractionated heparin was initially used and the patient was transitioned to complete a 6-month course of rivaroxaban on discharge. Fibrinolysis, thrombectomy, and surgery are reserved in severe case or refractory to anticoagulation. Duration of anticoagulation in mesenteric venous thrombosis provoked by diverticulitis is 6 months [5, 7]. Given the lack of a large trial, we propose that anticoagulation therapy should be individualized based on a case by case basis, carefully weighing the risks and benefits. Recurrent thrombosis may be as low as 0% to 3% in patients who received anticoagulation with most recurrences occurring within the first 30 days after presentation [12].

Colonoscopy remains paramount to exclude colorectal cancer. Timing of colonoscopy is generally advised at least 6 weeks after an episode of acute diverticulitis due to the risk of perforation or bleeding during an acute diverticular flare.
